# Effect of source and level of forage in the diet on in vitro ammonia emission from manure of Holstein and Jersey dairy cows

**DOI:** 10.3168/jdsc.2020-0012

**Published:** 2020-12-11

**Authors:** M.E. Uddin, M.A. Wattiaux

**Affiliations:** Department of Dairy Science, University of Wisconsin-Madison, Madison 53706

## Abstract

Reducing overall reactive N losses from dairy production systems depends substantially on reducing the atmospheric emission of manure ammonia (NH_3_). The objective of this study was to determine potential NH_3_-N emission of reconstituted manure using an in vitro protocol. Feces and urine were collected from a companion study designed as a Latin square in which 4 Holstein and 4 Jersey cows were fed diets containing 2 levels of forage neutral detergent fiber (NDF) [low-forage NDF (19%) vs. high-forage NDF (24%; dry matter basis)] from either alfalfa silage or corn silage (70:30 vs. 30:70 ratio of alfalfa silage NDF:corn silage NDF) arranged as a 2 × 2 factorial. All diets contained similar levels of crude protein (17%) and starch (23%), and had forage-to-concentrate ratios of 55:45 and 68:32 for low- and high-forage NDF diets, respectively. Measurements of NH_3_-N emission were conducted in a laboratory-scale chamber with 16 g of reconstituted manure (urine plus feces) incubated for 48 h at 15°C with sampling at 1, 3, 6, 12, 24, 36, and 48 h. Hourly NH_3_-N emissions data were analyzed using a repeated-measures mixed model in R (https://www.r-project.org/). The fixed effects were breed, forage NDF level, forage NDF source, time of sampling, and all possible interactions; cow was included as a random term. The cumulative 48-h NH_3_-N emissions and the scaled-up emissions accounting for daily output of manure from each cow were analyzed using the same model but without time of sampling. Level and source of forage in the diet tended to influence the pattern in hourly rate and 48-h cumulative emission, respectively. Accounting for daily manure volume differences, low-forage NDF diets led to lower estimates of daily NH_3_-N emissions than high-forage NDF diets (20% on a cow basis, 15% on a raw manure basis, and 18% on a manure-N basis). Compared with Holsteins, Jerseys emitted 17% lower estimated NH_3_-N on a cow basis, mainly due to lower manure excretion but tended to emit 15% more NH_3_-N expressed on a manure-N basis. Findings of this study suggested that cow breed and dietary forage NDF level should be considered in the prediction of NH_3_-N emission from the dairy industry.

## Highlights

• Effects of cow breed and dietary forage on manure ammonia-N emission were assessed
• Forage level and source affected hourly rate and cumulative emission, respectively
• Estimated daily emission was 20% lower for cows fed low-forage versus high forage
• Jerseys had 17% lower estimated daily manure ammonia-N emission than Holsteins
• Differences were due to variation in amount and ratio of fecal-to-urinary N excreted.

## Graphical Abstract

**Figure:**
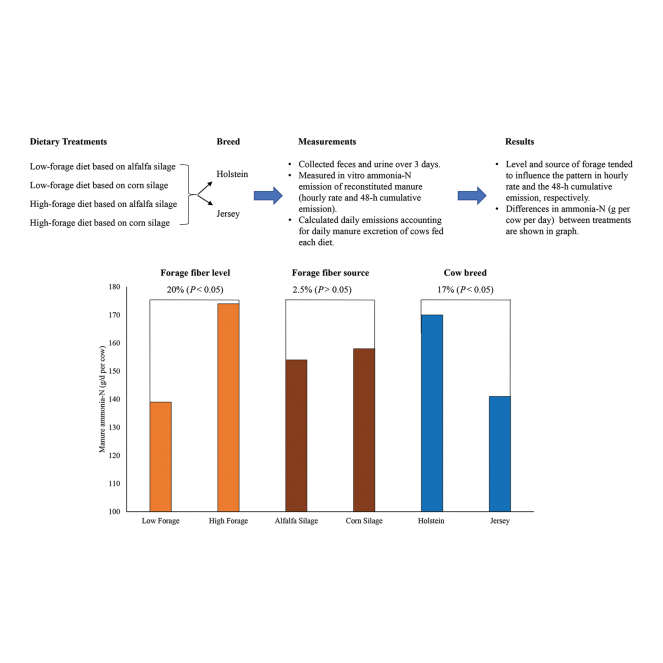
**Summary:** In this study, we determined the carry-over effects of cow breed, dietary forage level, and dietary forage source on ammonia-N emission of reconstituted manure (feces plus urine without bedding materials) using an in vitro protocol. Accounting for daily manure excretion differences, low-forage diets [19% forage neutral detergent fiber (NDF) in dietary dry matter] led to 20% lower estimated daily ammonia-N emissions than high-forage diets (24% forage NDF), whereas forage source (alfalfa silage vs. corn silage) did not affect manure ammonia-N emission. Compared with Holsteins, Jerseys emitted 17% less ammonia-N on a per cow per day basis (16% less on a metabolic body weight basis) mainly due to lower manure excretion. Findings of this study suggest that cow breed and dietary forage level should be considered in the prediction of ammonia-N emission from the dairy industry.

Dairy cattle have poor N utilization efficiency (i.e., conversion of feed N into milk N), which typically ranges from 25 to 35% (Hassanat et al., 2013; Arndt et al., 2015). The remaining feed N is excreted almost equally via feces and urine, although the proportion mostly depends on CP level and ratio of RDP to RUP in the diet (Olmos-Colmenero and Broderick, 2006). The excreted N is lost at each stage of the manure management chain (e.g., during collection, storage, and after land application of manure) in several forms; namely, ammonia (NH_3_), nitrous oxide, and nitrate (Wattiaux et al., 2019). Ammonia volatilization is the major form of N losses to the environment and accounts for 15 to 50% of the excreted manure N (Velthof et al., 2012). In a case study of concerns specific to the northeast dairy region of the United States, Rotz et al. (2020) estimated that NH_3_ emission was the greatest concerns for the Pennsylvania dairy industry because it was responsible for more than half of the state's emissions. The same study also suggested focusing on NH_3_ abatement to reduce overall reactive N (e.g., NH_3_, nitrous oxide, nitrate, and other forms of gaseous nitrogen oxides) losses from the dairy production system.

Ammonia released into the atmosphere forms particulate matter less than 2 µm in size, which might affect human health (Erisman and Schaap, 2003). Upon redeposition, NH_3_ can cause acid rain and soil acidification, eutrophication of aquatic ecosystems, and biodiversity loss (Bobbink et al., 1998). Additionally, NH_3_ is an indirect source of nitrous oxide, a potent greenhouse gas (Schreiber et al., 2012; Wattiaux et al., 2019). Furthermore, NH_3_ emission represents a loss of manure N that would otherwise be available for crop uptake upon land application.

Among NH_3_ abatement strategies, acidification was reported as having the greatest effect, whereas dietary manipulation was the most cost-effective way to reduce emissions from the dairy manure chain (Zhang et al., 2019). Among dietary strategies, reduction of dietary CP is the most effective way to abate emissions due to lowering urinary N relative to fecal N (Weiss et al., 2009; Aguerre et al., 2010) because urinary N is the main substrate for NH_3_ formation from manure (Weiss et al., 2009). Other potential dietary manipulations include changing the ratio of RDP to RUP (Davidson et al., 2003) and increasing the concentrate (van der Stelt et al., 2008) or starch level (Aguerre et al., 2011). Furthermore, increasing proportion of corn silage (**CS**) at the expense of alfalfa silage (**AS**) in the forage portion of the diet linearly decreased manure N excretions and increased fecal-to-urinary N urine ratio (Hassanat et al., 2013; Arndt et al., 2015), which might help in reducing manure NH_3_ emissions. However, Weiss et al. (2009) reported an increase in manure NH_3_ emissions when AS was replaced with CS. The reason for this controversial result might be a confounding effect associated with varying level of starch in the diet. Recently, we evaluated the effects of replacing AS with CS at 2 levels of forage NDF on cow performances, feces and urine output, and fecal and urinary N excretions in Holsteins and Jerseys, while maintaining similar starch, total NDF, and CP across diets (Uddin et al., 2020a). We found that low-forage NDF (**LF**)-fed cows excreted 17% less urinary N (expressed as percent of N intake) than high-forage NDF (**HF**)-fed cows. In addition, CS-fed cows tended to excrete 6% less manure N (expressed as percent of N intake) than AS-fed cows, along with an increase in feces-to-urine ratio (2.21 vs. 1.65). Thus, based on results of this recent study, we hypothesize herein that manure NH_3_ emission is lower for LF-fed than HF-fed cows and lower for CS-fed than AS-fed cows. Additionally, we expected a difference in daily manure NH_3_ emissions between Holsteins and Jerseys due to differential total manure N excretions but we did not expect a difference in manure NH_3_ emissions per unit of manure because both breeds had similar N excretions expressed as percent of N intake. Therefore, the objective of this study was to determine the effect of iso-nitrogenous and iso-starch diets with varying levels and sources of forage NDF on in vitro NH_3_-N emissions from manure of Holstein and Jersey cows.

The manure used in this study was collected from a companion study in which we describe dietary treatments in detail and report cow performance, manure production, and manure N excretion data (Uddin et al., 2020a). An institutional animal care and use committee–approved protocol was followed for animal use and care during manure collection, which was conducted at the Dairy Cattle Center, University of Wisconsin-Madison. Briefly, 4 primiparous Holstein and 4 primiparous Jersey cows were fed 4 diets arranged in a 2 × 2 factorial as split-plot 4 × 4 Latin square design with breed as main plot and diets as sub-plots. The dietary factors were forage NDF level [19.0 (LF) and 24.0% (HF), DM basis] and forage NDF source (70:30 and 30:70 ratio of AS NDF:CS NDF). Dietary treatments were offered as TMR with similar levels of CP (17%), starch (23%), and NE_L_ (1.5 Mcal/kg of DM). However, to keep the total dietary NDF similar between forage NDF levels and forage NDF source, nonforage NDF (mainly from soyhulls) was greater in LF than in HF diets, and the ratio of soybean meal to corn grain was lower in AS than in CS diets. Each Latin square period lasted 4 wk (3 wk of dietary adaptation followed by sampling during wk 4). Manure samples used for this study were from total collection of feces and urine (without acidification) conducted in period 3. Every 8 h of 3 consecutive days, the weight of feces and urine were recorded and approximately 500 g of feces and 100 mL of urine were collected after following a hand-mixing procedure. The 9 fecal and 9 urine samples collected from each cow were composited and stored separately at −20°C until further analysis. The detailed chemical composition of manure is reported in Uddin et al. (2020b).

Manure NH_3_ emission from 8 composite samples from 8 cows was determined in triplicate (3 runs using 3 sub-samples) over a 48-h measurement period using a laboratory-scale ventilated chamber. The chamber details, including construction of the chamber and calibration, have been described in Misselbrook et al. (2005) and later in Powell et al. (2011b). Briefly, chambers were constructed with plastic drainage pipe (10 cm in diameter and 19 cm high). The base of the pipe was capped permanently with glue, and a top lid was made in such a way that it could be fitted with silicone grease to ensure a proper seal. Each lid had 4 inlet and 4 outlet ports to allow proper air mixing inside the chamber. One acid trap (0.075 L, 0.02 mol L^−1^ of orthophosphoric acid) was connected to the inlet to remove any NH_3_ coming through the inlet air, and a second acid trap was connected to outlet to collect NH_3_ during incubation of manure sample. An entire setup of 4 chambers was installed in a large temperature-regulated incubator (15°C). As the incubation temperature, we chose average Wisconsin temperature (15°C) across 3 seasons of spring, fall, and winter because a study conducted under Wisconsin condition showed maximum NH_3_-emitting potential of manure at or close to this temperature (Powell et al., 2008). In each chamber, approximately 16 g of reconstituted manure was incubated on a petri dish. The reconstitution was done to maintain the same feces-to-urine ratio as produced by each cow (wet weight basis), as reported in Uddin et al. (2020a). Upon thawing and proper mixing, the required amount of feces was placed first in a petri dish, which was temporarily covered with parafilm until addition of urine. When ready, the required amount of urine was then poured on the petri dish and mixed properly with fecal material. Immediately thereafter, the petri dishes were placed in a chamber, which was sealed with a greased lid. Each chamber was then connected at the inlet and outlet ports. The airflow was maintained at 4 L min^−1^ and the outlet acid trap was changed after 1, 3, 6, 12, 24, 36, and 48 h. The outlet acid was diluted to 0.1 L with deionized water and the diluted solution was analyzed for NH_4_^+^ with a flow injection analyzer (Lachat Instruments, Loveland, CO; QuikChem Method 12-107-06-2-A). The amount (mg) of NH_3_-N collected in the acid trap was calculated as the product of NH_4_^+^ N concentrations in acid trap solution (mg L^−1^) and the volume of acid trap solution (0.01 L). Hourly rate of NH_3_-N emission was calculated by dividing the amount of NH_3_-N from the acid trap solution by the hours it remained connected to the chamber. The cumulative NH_3_-N emission for each treatment was calculated by summing emission of all time points measured over the 48 h of measurement.

Although laboratory-scale measurements capture the effects of manure composition on NH_3_ emission, they do not account for differences in daily output of manure among treatments, as observed in Uddin et al. (2020a). Results presented in Uddin et al. (2020a) indicated that cow breed and dietary forage NDF affected feces-to-urine ratio, daily excretion of fecal and urinary N, and fat- and protein-corrected milk (**FPCM**) production. Therefore, we calculated manure NH_3_-N emissions adjusted for daily manure volume, FPCM, and manure N excretion, and we called these variables the “scaled-up” variables expressed in the following ways: grams of NH_3_-N/cow per day, grams of NH_3_-N/kilogram of FPCM, grams of NH_3_-N/ kilogram of raw manure, and NH_3_-N as percentage of total manure N excreted.

Use of manure from only period 3 of the companion study precluded us from analyzing the data as a Latin square. However, our data included 4 observations per breed, per forage NDF level, and per forage NDF source, as well as 3 in vitro incubation replicates. Thus, hourly NH_3_-N emission was analyzed using the lme function of lme4 package in R version 3.5.3 (https://www.r-project.org/) using the repeated-measure mixed effects model containing fixed effects of cow breed, forage NDF level, forage NDF source, sampling time, and all 2- and 3-way interactions among forage NDF level, forage NDF source, and sampling time. The cow was fitted as random effect and the auto-correlation covariance structure, with sampling time as continuous covariate, was fitted using the corCAR1 function. Inclusion of in vitro incubation replicates within cow as random effect did not improve the model, and thus they were dropped from the final model. Cumulative NH_3_-N emission and scaled-up NH_3_-N emission variables calculated for a 48-h period of incubation were analyzed using a simple nonrepeated mixed model containing fixed effects of cow breed, forage NDF level, forage NDF source, interaction between forage NDF level × forage NDF source, and random effect of cow. Effects were reported as significant or as tendency for *P* ≤ 0.05 and 0.05 < *P* ≤ 0.10, respectively.

The hourly rates of NH_3_-N emission (Figure 1) were within the range of those reported in previous studies (Powell et al., 2011a,b). Hourly NH_3_-N emissions peaked around 24 h after starting incubation and decreased thereafter, returning to the initial (1-h) value after 48 h of incubation. In this study, hourly manure NH_3_-N emission did not differ between breeds or levels and sources of dietary forage NDF. However, we detected a significant interaction between forage NDF level and sampling time. The hourly rate of manure NH_3_-N emission was similar for the first 6 h of incubation, whereas subsequent emissions were consistently higher for manure from the HF-fed cows than from the LF-fed cows (Figure 1). Compared with that of HF-fed cows, manure from LF-fed cows tended (*P* < 0.10) to emit less NH_3_-N, particularly around peak emission hours (12 and 24 h; Figure 1). This tendency might be associated with the effect of forage NDF level on concentrations of manure N as a percentage of manure DM (Uddin et al., 2020b). The significant effect of sampling time on manure NH_3_-N emission (observed both for breed and dietary treatments) in this study is important for the timing of manure processing or treatment (e.g., manure acidification, solids-to-liquid separation of manure) when aiming to reduce manure NH_3_-N emission. Other studies have also reported that most NH_3_-N was lost within 36 h after mixing feces with urine (Holly et al., 2017).

**Figure 1:**
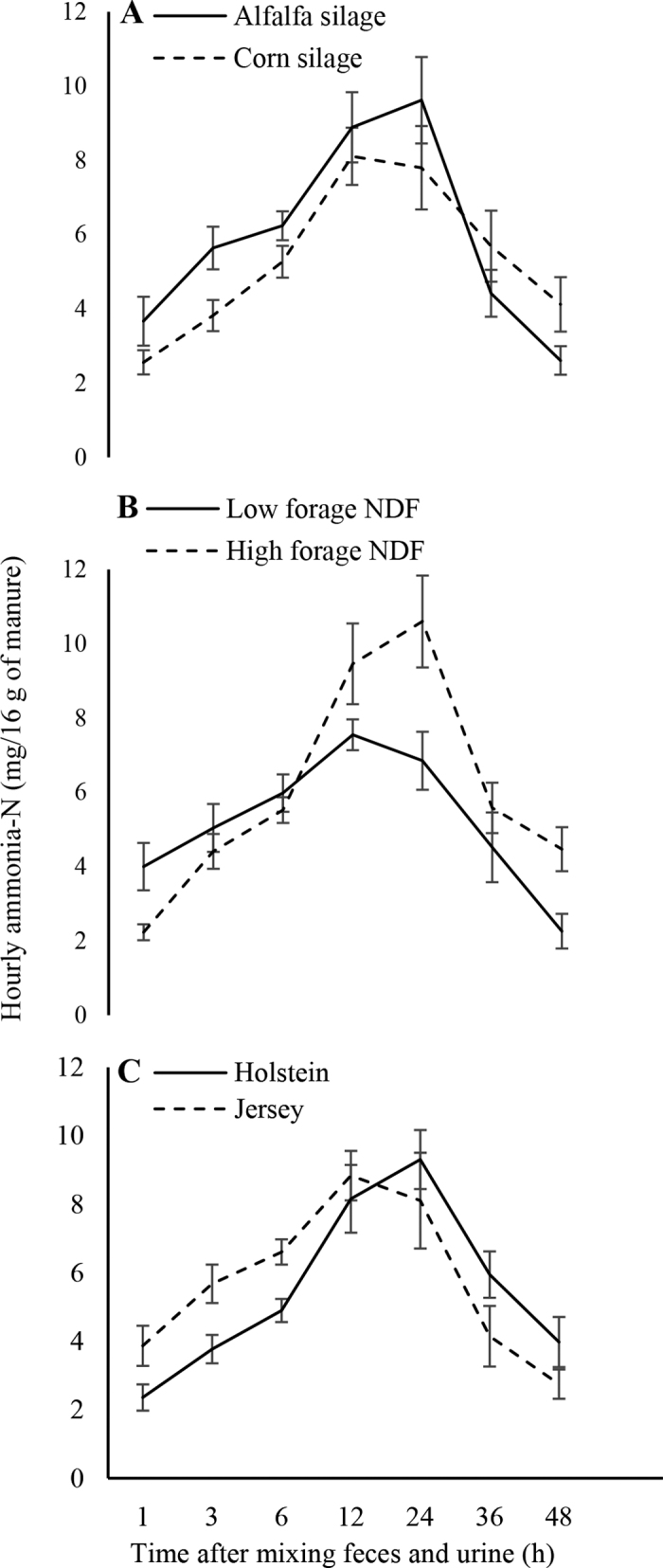
Hourly ammonia-N emissions (mean ± SE) from 16 g of manure incubated over a 48-h period as affected by (A) dietary forage NDF source, (B) dietary forage NDF level, and (C) cow breed (*P*-value: forage NDF source = 0.74, forage NDF level = 0.09, cow breed = 0.65, hour <0.01, forage NDF level × forage NDF source = 0.41, forage NDF level × hour <0.01, forage NDF source × hour = 0.08, forage NDF level × source × hour <0.01).

Cumulative NH_3_-N emissions did not differ between cow breeds and forage NDF levels (Figure 2), again likely due to similar concentrations of total N and ammoniacal N [NH_4_^+^ + NH_3,aq_ (aqueous)] in manure (Uddin et al. 2020b). However, CS-fed cows tended to emit less cumulative manure NH_3_-N than AS-fed cows (Figure 2). The greater feces-to-urine ratio (2.21 vs. 1.65) for CS-fed cows than for AS-fed cows (Uddin et al. 2020a) might have contributed to this effect because urea in the urine is the primary source of volatilized NH_3_ (James et al., 1999). Nevertheless, cumulative NH_3_-N emission pattern and magnitude were comparable to the values reported by Powell et al. (2011a) for a 16.8% CP diet, which is similar to the 17.0% (DM basis) average CP content of our dietary treatments.

**Figure 2:**
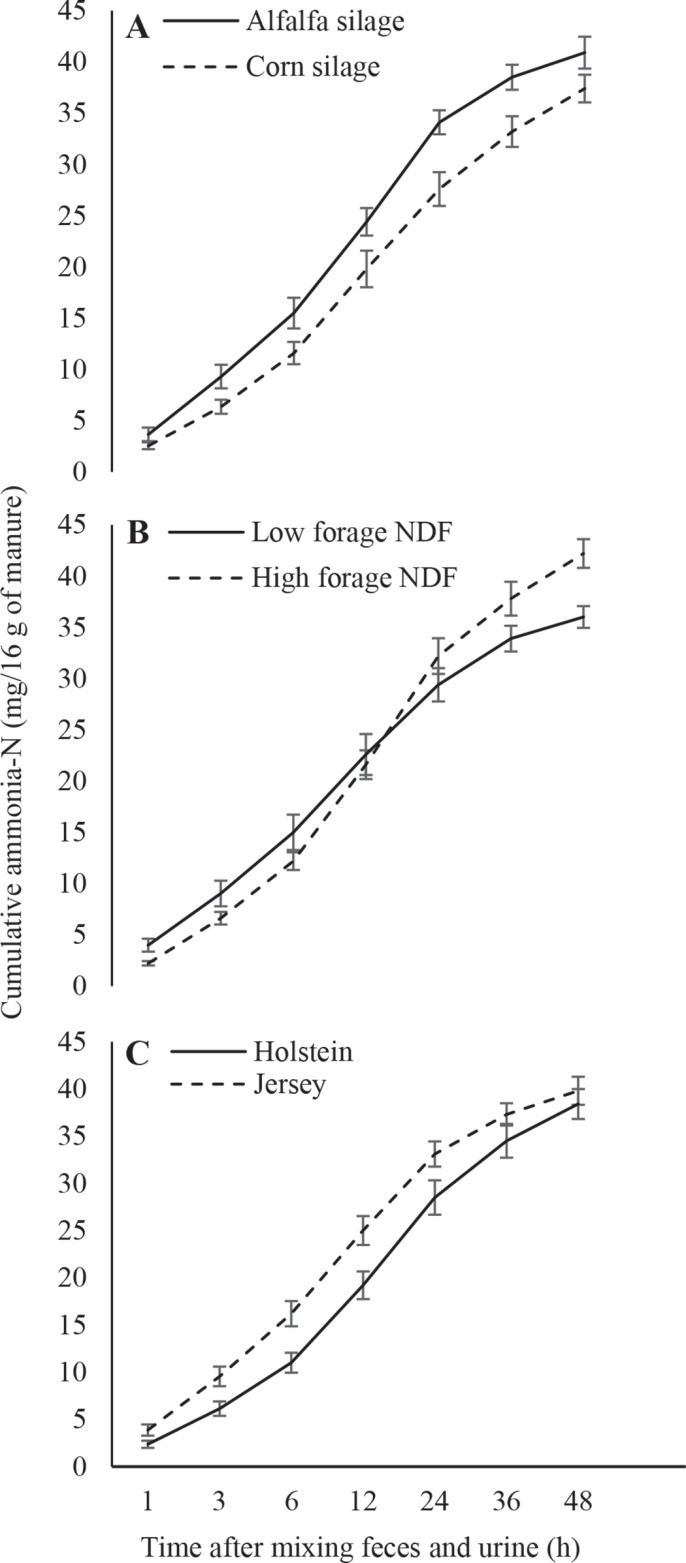
Cumulative ammonia-N emissions (mean ± SE) from 16 g of manure incubated over a 48-h period as affected by (A) dietary forage NDF source, (B) dietary forage NDF level, and (C) cow breed (*P*-value: forage NDF level = 0.18, forage NDF source = 0.05, cow breed = 0.10, hour <0.01, forage NDF level × forage NDF source = 0.64, forage NDF level × hour <0.01, forage NDF source × hour = 0.01, forage NDF level × forage NDF source × hour = 0.22).

Results of the scaled-up variables (g of NH_3_-N/cow per day, g of NH_3_-N/kg of FPCM, g of NH_3_-N/kg raw manure, and NH_3_-N as % of total manure N excreted) calculated by accounting for daily output of manure, FPCM, and manure N are presented in Table 1. Only main effects are presented because none of the interactions were significant. Accounting for daily manure output, LF-fed cows would emit 20% less NH_3_-N (g/cow per day) than HF-fed cows. In agreement with our findings, van der Stelt et al. (2008) also reported an increase in manure NH_3_-N emissions on a per cow basis when NDF in the diet was increased. This effect in our study might be associated with the lower excretion of daily urinary N for LF-fed cows than for HF-fed cows (Uddin et al., 2020a), because urinary N has greater potential to be volatilized than fecal N (James et al., 1999). This greater proportion of urinary N excretion relative to fecal N also increased N volatilization for HF-fed than for LF-fed cows when expressed either on a per kilogram of manure or as percentage of manure N basis (Table 1). Thus, increasing level of concentrate in the diet (or replacing forage NDF with nonforage NDF, mainly from soyhulls in this case) not only helped to reduce N excretion in manure but potentially reduced manure NH_3_-N emissions during storage. We detected a tendency for greater NH_3_-N as percentage of manure N for AS-fed cows than for CS-fed cows but no main effect of forage NDF source on scaled-up NH_3_-N emission variables (Table 1). In contrast, Weiss et al. (2009) reported a reduction in manure NH_3_-N emissions when cows were fed diets containing a greater proportion of AS than CS. Unlike the current study, in which starch content was similar across dietary treatments, there was a greater starch content in the AS-based diet than in the CS-based diet in the study of Weiss et al. (2009). In the case of cow breed, Jerseys as expected, emitted less NH_3_-N (17 and 16% when expressed as g/cow per day and g/kg of metabolic BW, respectively; Table 1) than Holsteins, mainly due to the differential manure volume (56 vs. 72 kg of raw manure/cow per day for Jersey and Holstein, respectively; Uddin et al., 2020a). However, compared with Holsteins, Jerseys tended to emit 15% greater NH_3_-N when this was expressed as percentage of excreted manure N. This greater value was associated with a lower feces-to-urine ratio for Jerseys than for Holsteins (Uddin et al., 2020a). However, manure NH_3_-N intensity (g of NH_3_-N/kg of FPCM) was not affected by any treatments.

**Table 1:** Effects of cow breed, dietary forage NDF level, and dietary forage NDF source on ammonia-N emission

Ammonia-N emissions^1^	Treatment diet^2^					Breed			*P*-value		
	LF		HF		SEM	Holstein	Jersey	SEM	Forage NDF		Breed
	AS	CS	AS	CS					Level	Source	
NH_3_-N (g/cow per day)	134	143	174	173	7.860	170	141	5.560	0.03	0.60	0.02
NH_3_-N (g/kg of metabolic BW)	1.36	1.35	1.66	1.54	0.078	1.60	1.35	0.055	0.05	0.44	0.04
NH_3_-N (g/kg of FPCM)	5.14	5.24	6.05	5.30	0.357	5.10	5.80	0.252	0.25	0.45	0.15
NH_3_-N (g/kg of raw manure)	2.30	2.20	2.80	2.50	0.103	2.40	2.49	0.073	0.03	0.11	0.45
NH_3_-N (% of manure N)	49.5	42.0	58.6	52.3	2.780	47.0	54.2	1.960	0.04	0.08	0.08

1 Ammonia-N emissions: Cumulative in vitro manure ammonia-N emissions measured over a 48-h period adjusted for daily output of manure from each cow (g/cow per day), per kilogram of metabolic BW, per daily production of fat- and protein-corrected milk (g/kg of FPCM), per kilogram of raw manure (g/kg of raw manure), and as a percentage of manure N excretion (% of manure N).

2 Treatment diets: LF = low-forage diet containing 19.0% forage NDF; HF = high-forage diet containing 24.0% forage NDF; AS = alfalfa silage–based diet with a 70:30 ratio of alfalfa silage NDF:corn silage NDF; CS = corn silage–based diets with a 30:70 ratio of alfalfa silage NDF:corn silage NDF.

In our study, feces and urine were collected separately, and manure was reconstituted by properly mixing them without inclusion of external organic or inorganic compounds. Furthermore, manure was incubated over 48 h at a constant temperature of 15°C. Therefore, our study conditions did not fully simulate the typical dairy manure handling scenario, where feces and urine are often mixed with bedding materials, lime, and wastewater, and manure is subjected to temperature variation during storage. The standardized protocol used here to detect treatment differences should be considered while interpreting and comparing our results with other studies that have assessed potential manure NH_3_-N emissions. Our study conditions, however, allowed us to capture the breed and dietary forage effects on manure NH_3_-N-emitting potential with minimal confounding effects. In summary, under our experimental conditions, cow breed and dietary forage NDF level and forage NDF source did not affect hourly or cumulative NH_3_-N emissions from 16 g of manure incubated over a 48-h period. However, when accounting for daily manure volume and manure N excretions, cow breed and forage NDF level did affect estimated manure NH3-N emissions measured over 48 h from the daily output of manure from each cow.

## References

[bib1] Aguerre M.J., Wattiaux M.A., Hunt T., Larget B.R. (2010). Effect of dietary crude protein on ammonia-N emission measured by herd nitrogen mass balance in a freestall dairy barn managed under farm-like conditions. Animal.

[bib2] Aguerre M.J., Wattiaux M.A., Powell J.M., Broderick G.A., Arndt C. (2011). Effect of forage-to-concentrate ratio in dairy cow diets on emission of methane, carbon dioxide, and ammonia, lactation performance, and manure excretion. J. Dairy Sci..

[bib3] Arndt C., Powell J.M., Aguerre M.J., Wattiaux M.A. (2015). Performance, digestion, nitrogen balance, and emission of manure ammonia, enteric methane, and carbon dioxide in lactating cows fed diets with varying alfalfa silage-to-corn silage ratios. J. Dairy Sci..

[bib4] Bobbink R., Hornung M., Roelofs J.G.M. (1998). The effects of air-borne nitrogen pollutants on species diversity in natural and semi-natural European vegetation. J. Ecol..

[bib5] Davidson S., Hopkins B.A., Diaz D.E., Bolt S.M., Brownie C., Fellner V., Whitlow L.W. (2003). Effects of amounts and degradability of dietary protein on lactation, nitrogen utilization, and excretion in early lactation Holstein cows. J. Dairy Sci..

[bib6] Erisman J.W., Schaap M. (2003). The need for ammonia abatement with respect to secondary PM reductions in Europe. Environ. Pollut..

[bib7] Hassanat F., Gervais R., Julien C., Massé D.I., Lettat A., Chouinard P.Y., Petit H.V., Benchaar C. (2013). Replacing alfalfa silage with corn silage in dairy cow diets: Effects on enteric methane production, ruminal fermentation, digestion, N balance, and milk production. J. Dairy Sci..

[bib8] Holly M.A., Larson R.A., Powell J.M., Ruark M.D., Aguirre-Villegas H. (2017). Greenhouse gas and ammonia emissions from digested and separated dairy manure during storage and after land application. Agric. Ecosyst. Environ..

[bib9] James T., Meyer D., Esparza E., Depeters E.J., Perez-Monti H. (1999). Effects of dietary nitrogen manipulation on ammonia volatilization from manure from Holstein heifers. J. Dairy Sci..

[bib10] Misselbrook T.H., Powell J.M., Broderick G.A., Grabber J.H. (2005). Dietary manipulation in dairy cattle: Laboratory experiments to assess the influence on ammonia emissions. J. Dairy Sci..

[bib11] Olmos-Colmenero J.J., Broderick G.A. (2006). Effect of dietary crude protein concentration on milk production and nitrogen utilization in lactating dairy cows. J. Dairy Sci..

[bib12] Powell J.M., Aguerre M.J., Wattiaux M.A. (2011). Tannin extracts abate ammonia emissions from simulated dairy barn floors. J. Environ. Qual..

[bib13] Powell J.M., Aguerre M.J., Wattiaux M.A. (2011). Dietary crude protein and tannin impact dairy manure chemistry and ammonia emissions from incubated soils. J. Environ. Qual..

[bib14] Powell J.M., Broderick G.A., Misselbrook T.H. (2008). Seasonal diet affects ammonia emissions from tie-stall dairy barns. J. Dairy Sci..

[bib15] Rotz C.A., Stout R.C., Holly M.A., Kleinman P.J.A. (2020). Regional environmental assessment of dairy farms. J. Dairy Sci..

[bib16] Schreiber F., Wunderlin P., Udert K., Wells G. (2012). Nitric oxide and nitrous oxide turnover in natural and engineered microbial communities: Biological pathways, chemical reactions, and novel technologies. Front. Microbiol..

[bib17] Uddin M.E., Larson R.A., Wattiaux M.A. (2020). Effect of dairy cow breed, and dietary forage on greenhouse gas emissions from manure during storage and after field application. J. Clean. Prod..

[bib18] Uddin M.E., Santana O.I., Weigel K.A., Wattiaux W.A. (2020). Enteric methane, lactation performances, digestibility, and metabolism of nitrogen and energy of Holsteins and Jerseys fed 2 levels of forage fiber from alfalfa silage or corn silage. J. Dairy Sci..

[bib19] van der Stelt B., van Vliet P.C., Reijs J.W., Temminghoff E.J., van Riemsdijk W.H. (2008). Effects of dietary protein and energy levels on cow manure excretion and ammonia volatilization. J. Dairy Sci..

[bib20] Velthof G.L., van Bruggen C., Groenestein C.M., de Haan B.J., Hoogeveen M.W., Huijsmans J.F.M. (2012). A model for inventory of ammonia emissions from agriculture in the Netherlands. Atmos. Environ..

[bib21] Wattiaux M.A., Uddin M.E., Letelier P., Jackson R.D., Larson R.A. (2019). Invited review: Emission and mitigation of greenhouse gases from dairy farms: The cow, the manure, and the field. Appl. Anim. Sci..

[bib22] Weiss W.P., Willett L.B., St-Pierre N.R., Borger D.C., McKelvey T.R., Wyatt D.J. (2009). Varying forage type, metabolizable protein concentration, and carbohydrate source affects manure excretion, manure ammonia, and nitrogen metabolism of dairy cows. J. Dairy Sci..

[bib23] Zhang N., Bai Z., Winiwarter W., Ledgard S., Luo J., Liu J., Guo Y., Ma L. (2019). Reducing ammonia emissions from dairy cattle production via cost-effective manure management techniques in China. Environ. Sci. Technol..

